# DPD Ultra‐Rapid Metabolizer Status and Efficacy of 5‐Fluorouracil Treatment: A Real‐World Study

**DOI:** 10.1111/fcp.70035

**Published:** 2025-07-07

**Authors:** Govind Kallee, Gérard Milano, Florence Duffaud, Laetitia Dahan, Joseph Ciccolini

**Affiliations:** ^1^ PRISM, Biogenopôle La Timone University Hospital of Marseille Marseille France; ^2^ COMPO, Inserm U1068 CRCM Marseille France; ^3^ Centre Antoine Lacassagne Nice France; ^4^ Medical Oncology La Timone University Hospital of Marseille Marseille France; ^5^ Digestive Oncology La Timone University Hospital of Marseille Marseille France

**Keywords:** 5FU, DPD, efficacy, safety

## Abstract

**Background:**

Anticancer drug 5FU is extensively metabolized by dihydropyrimidine dehydrogenase (DPD), an enzyme with high interindividual variability. Poor metabolizer (PM, i.e., DPD deficient) patients are at risk of life‐threatening toxicities. Whether ultra‐rapid metabolizer (UM) status could conversely compromise 5FU efficacy remains to be investigated.

**Methods:**

In this real‐world study, 352 adult patients treated with a 5FU‐containing regimen were screened. Patients were classified as normal (extensive metabolizer, EM), PM, or UM on DPD function based upon baseline plasma uracil monitoring. The impact of DPD status on efficacy and safety endpoints was investigated.

**Results:**

Patients were categorized on DPD as UM (11.9%), EM (75.9%), and PM (12.2%). The response rate was 54.5%, with median PFS and OS of 13.9 and 19 months, respectively. PM patients were treated with an average 13% lower 5FU starting dose. There was no statistical difference in efficacy between UM and other patients. Severe toxicities were observed in less than 5% of patients, an incidence significantly lower than commonly reported with 5FU‐containing regimen and was comparable between UM, EM, and PM patients. Our observations suggest that UM status is not associated with the lack of efficacy of 5FU. In addition, upfront DPD testing with adaptive dosing helps to reduce the incidence of severe toxicities, as PM patients on reduced doses did not have more severe toxicities than other patients treated with standard doses, while exhibiting similar efficacy in terms of response rate and survival.

**Conclusion:**

When upfront DPD screening with adaptive dosing is performed, no difference is observed between UM, EM, and PM patients in terms of efficacy and safety.

**Trial Registration:**

#PADSA3GKW7

**Abbreviations**
DPD: dihydropyrimidine dehydrogenase
EM: extensive metabolizer
EMA: European Medical Agency
LOQ: limit of quantification
OS: overall survival
PFS: progression‐free survival
PM: poor metabolizer
UM: ultra‐rapid metabolizer
5FU: 5‐fluorouracil

## 1 Background

5FU (5FU) is widely used in oncology and is extensively metabolized by the liver enzyme dihydropyridine dehydrogenase (DPD) [1]. DPD deficiency can cause fatal toxicity after standard doses [2]. Patients with impaired DPD (identified using either genotyping or phenotyping techniques) are expected to exhibit reduced drug clearance [3, 4, 5, 6], thus being overexposed and prone to life‐threatening toxicities. Several methods have been proposed to determine DPD status in patients [7, 8, 9]. Since endogenous plasma uracil is converted to dihydrouracil by DPD, baseline plasma uracil is considered as a surrogate marker for DPD activity. In 2018, the French health authorities made such uracil check mandatory for screening DPD deficiency [10] before starting 5FU or oral capecitabine with dose reduction if required in poor metabolizer (PM) patients and contra‐indication in patients with complete deficiency. The threshold for DPD deficiency is U > 16 ng/mL: the higher the U, the more severe is expected to be the deficiency and the lower should thus be the dosing, [11, 12] although to direct correlation was evidence between U plasma levels and 5FU clearance [13]. Patients with U > 150 ng/mL are considered with a complete DPD deficiency precluding 5FU or capecitabine. Others such as the European Medical Agency (EMA), the UK National Health System, or the Dutch Pharmacogenetics Working Group, have released recommendations for DPD testing, but have not made it mandatory thus far [14].

The impact of ultra‐rapid metabolizer (UM) status on 5FU efficacy is yet to be determined, as very few studies have evaluated the link between UM patients and efficacy of fluoropyrimidine drugs. A single clinical report has suggested that patients with elevated DPD activity could exhibit worse progression‐free survival (PFS), overall survival (OS), and response rates [15]. Recently, it was shown that patients bearing the *DPYD* rs4294451‐T allele carriers with increased DPD expression in the liver have a shorter OS as compared with wild‐type patients [16]. Here, we present a real‐world study in which we compared the efficacy of 5FU‐containing regimens in patients stratified according to their DPD status, with a priori dose reduction in PM patients. In addition, we evaluated and compared differences in dosing and the incidence of severe toxicities among the patients, depending on their DPD status.

## 2 Patients and Methods

This is a retrospective, non‐interventional, observational study conducted at the University Hospital of Marseille, Marseille, France. The main objective was to compare the efficacy of the treatment between UM and non‐UM patients. The secondary objective was to compare the severe toxicities observed among the patients, depending on their DPD status. This study was approved by our Review Board (Cellule d'Evaluation at the Direction de la Recherche Clinique & de l'Innovation (DRCI), APHM Marseille France) and was conducted in accordance with the European recommendations regarding the Clinical Trials Regulation (EU) 536/2014 and the General Data Protection Regulation (GDPR; EU) 2016/679. This study has been granted French MR004 legal status for non‐interventional studies and registered as PADS#WJ8VD8. Due to the non‐interventional nature of the study and the fact that all patients were treated according to standard French guidelines with no additional sampling or examinations, the review board waived informed consent.

Upfront DPD status determination: DPD was evaluated through the measurement of plasma uracil levels [17]. Uracil was measured by UPLC‐UV using a EMA‐, ISO15189‐validated technique, with a Limit of Quantification (LOQ) of 5 ng/mL and a precision and accuracy both below 15% [18]. Patients were next classified as “ultrarapid metabolizer” (UM: uracilemia < 5 ng/mL LOQ); “extensive metabolizer” (EM: 5 ng/mL ≤ uracilemia ≤ 16 ng/mL), “poor metabolizer” (PM: 16 ng/mL < uracilemia < 150 ng/mL), and with complete DPD deficiency (uracilemia ≥ 150 ng/mL).

### 2.1 Data Collection

Demographic, clinical, and biological data were collected retrospectively from patients' records stored in the institution database. Efficacy was measured according to standard RECIST evaluation. PFS was defined as the time to progression or death from any cause. OS was defined as the time from the start of treatment that patients are still alive. Side effects were graded according to the CTCAE version 4.0. All patients were treated according to validated protocols in their respective indications, with possible upfront dose adjustments due to comorbidities or poor performance status. Due to the variety in tumor types and regimen was a limitation to this study, a global analysis was first made, followed by an analysis focusing only on the subset of patients with metastatic colorectal cancer (mCRC).

### 2.2 Data Analysis

Correlations of variables with DPD status were determined using a Chi‐squared test or an exact Fisher test. The comparison of continuous variables was conducted using one‐way analysis of variance (ANOVA), Welch's ANOVA, and the Kruskal–Wallis test; the appropriate statistical test was selected based on the distribution of the variables.

Alpha risk was set at 5% and two‐tailed tests were used. PFS and OS curves were constructed using the Kaplan–Meier method and compared using the log‐rank test. Differences were assessed by the log‐rank test. A two‐sided *p* value of less than 0.05 was considered statistically significant. These analyses were performed using Python V3.11.0. To limit the impact of confounding factors, the results were adjusted using multivariate analyses, considering variables with the potential to impact efficacy and survival markers. A multivariate logistic regression was performed to assess the relationship between initial 5FU dose and the explanatory variables: age, gender, body mass index (BMI), performance status, DPD status, renal failure, and cancer stage. A second multivariate logistic regression was performed to assess the relationship between treatment toxicity and the explanatory variables: gender, age, BMI, performance status, renal insufficiency, DPD status, stage of disease, pretreatment with 5FU, monotherapy, doublet or triplet chemotherapy, initial dose of 5FU, reduction of 5FU dose during treatment, and cumulative dose of 5FU. A third multivariate linear regression was performed to assess the relationship between response and the explanatory variables: gender, age, performance status, DPD status, stage of disease, single agent chemotherapy vs. others, initial 5FU dose, cumulative 5FU dose, and toxicity to therapy. A fourth multivariate linear regression was performed to assess the relationship between PFS and the explanatory variables: gender, age, performance status, DPD status, stage of disease, single agent chemotherapy vs. other, initial 5FU dose, cumulative 5FU dose, and tolerability. A final multivariate linear regression was performed to assess the relationship between OS and the explanatory variables: gender, age, performance status, DPD status, disease stage, single agent chemotherapy vs. others, and cumulative 5FU dose. Data were analyzed for multicollinearity using the Belsley–Kuh–Welsch technique. Heteroskedasticity and normality of residuals were assessed using the White and Shapiro–Wilk tests, respectively. A *p*‐value of 0.05 was considered statistically significant, and patients with missing data were excluded from the analysis. Statistical analyses were performed using EasyMedStat (version 3.31; www.easymedstat.com).

## 3 Results

In January 2023, we monitored 352 adult patients receiving 5FU for a solid tumor and hospitalized between 01/01/2020 and 09/15/2022 at the University Hospitals of Marseille. All patients were treated following current guidelines, including baseline DPD testing per French recommendations.

### 3.1 Patients' Characteristics at Baseline

Mean/median uracilemia was 12.7/10 ng/mL (range: < 5–97 ng/mL). Patients were subsequently categorized as UM (11.9%), EM (75.9%), and PM (12.2%) on their DPD status. Tumor localization was mainly gastrointestinal (82.4%: colon [40.9%], pancreas [22.2%], stomach [10.8%], and esophagus [8.5%]). Other tumor types were head and neck in 14.2% of patients and various localizations for the remaining 3.4% of patients. There was no statistical difference in the baseline characteristics of patients according to their DPD status. Patient characteristics are shown in Table 1.

**TABLE 1:** Patient characteristics at baseline.

	UM	PM	EM	Total	*p*
Number	42	43	267	352	
Sex ratio M/F	47.6%/52.4%	67.4%/32.6%	58.4%/41.6%	57.7%/42.3%	0.127
Mean age (years) (± standard deviation)	64.9 ± 11.05	65.03 ± 11.85	64.17 ± 12.03	64.4 ± 11.38	0.769
≥ 70 years	33.3%	39.5%	34.1%	34.6%	0.884
Mean BMI (kg/m^2^) (± standard deviation)	23.31 ± 4.06	24.02 ± 3.96	23.37 ± 4.86	23.37 ± 4.68	0.387
Mean body surface area (m^2^) (± standard deviation)	1.73 ± 0.214	1.78 ± 0.185	1.74 ± 0.216	1.74 ± 0.204	0.311
Performance status					
0 or 1	61.91%	60.46%	66.29%	65.05%	0.63
≥ 2	38.09%	39.54%	33.71%	34.95%	
% patients with chronic renal failure (GRF < 60 ml/min/1.73 m2)	4.8	9.3	4.9	5.4	0.097
Tumor type (%)					0.237
Colorectal	30.9%	58.2%	39.7%	40.9%	
Pancreas	23.8%	20.9%	22.1%	22.2%	
ORL	21.4%	11.7%	13.6%	14.2%	
Stomach	4.8%	4.6%	12.7%	10.8%	
Esophagus	11.9%	2.3%	9.0%	8.5%	
Unknown	4.8%	2.3%	1.1%	1.7%	
Bladder	2.4%	0%	0.7%	0.8%	
Gynecologist	0%	0%	0.7%	0.6%	
Cutaneous	0%	0%	0.4%	0.3%	
Metastases (%)					0.327
None	50%	58.2%	50.2%	51.1%	
Unvisceral	35.7%	13.9%	24.7%	24.7%	
Multiviscérales	14.3%	27.9%	25.1%	24.2%	
Histology					0.38
Adenocarcinoma	64.4%	72.1%	70.4%	69.9%	
Squamous cell cancer	33.3%	23.3%	28.1%	28.1%	
Endocrine tumor	2.3%	4.6%	1.5%	2.0%	
Pretreatment with chemotherapy (%)					
No	92.9%	86.0%	87.6%	88.1%	
Yes without 5FU	2.3%	4.6%	8.3%	7.1%	0.095
Yes with 5FU	4.8%	9.4%	4.1%	4.8%	0.135

### 3.2 DPD Status and Drug Regimen

Treatment was neoadjuvant in 15.7% (19.0%; 15.3%; 13.9% in UM, EM, and PM, respectively, *p* > 0.05, n.s.); adjuvant in 40.0% (35.7%; 40.1%; 44.3% in UM, EM, and PM, respectively, *p* > 0.05); first line in 37.8% (35.7%; 38.6%; 34.9% in UM, EM, and PM, respectively, *p* > 0.05); second line in 4.3% of cases (4.8%; 4.2%; 4.6% in UM, EM, and PM, respectively, *p* > 0.05); third line and other in 2.2% of cases (4.8%; 1.8%; 2.3% in UM, EM, and PM, respectively, *p* > 0.05). To facilitate data processing, treatments were classified as mono‐, duet, or triplet chemotherapy. Further association with biologics (monoclonal antibodies), and/or immunotherapy was also included (Table 2). We examined whether DPD status led to differences in treatment. Patients were treated with a single agent in 11.7% of cases (9.8%; 10.3%; 20.9% in UM, EM, and PM, respectively); 9.1% alone, 1.7% in combination with monoclonal antibody, and 0.9% with endocrine therapy. Patients received doublet chemotherapy in 62.5% of cases (69.0%; 62.9%; 53.5% in UM, EM, and PM, respectively); 37.5% without monoclonal antibody or immunotherapy, 17.3% with monoclonal antibody, and 7.7% with immunotherapy. The remaining patients (25.8%) were treated with triplet chemotherapy (21.2%; 26.8%; 25.6% in UM, EM, and PM, respectively); 24.3% alone; 1.5% combined with monoclonal antibody.

**TABLE 2:** Treatment types.

Protocol name	Molecules	Classification
5FU	5FU	Single agent
5FU Bevacizumab	5 FU Bevacizumab	Single agent and monoclonal antibody
5FU Dacarbazine	5FU Dacarbazine	Single agent and endocrine therapy
5FU platinum salts	5FU platinum salts	Double chemotherapy
FOLFOX	Folinic Acid 5FU Oxaliplatin	Double chemotherapy
FOLFIRI	Acide folinic 5FU Irinotecan	Double chemotherapy
FUMIR	5FU Mitomycine	Double chemotherapy
RAPIDO	FOLFOX	Double chemotherapy
5FU platinum salts Cetuximab	5FU platinum salts Cetuximab	Double chemotherapy and monoclonal antibody
FOLFOX or FOLFIRI Herceptin	Folinic acid 5FU Oxaliplatin or Irinotecan Herceptin	Double chemotherapy and monoclonal antibody
FOLFOX or FOLFIRI Bevacizumab	Folinic acid 5FU Oxaliplatin or Irinotecan Bevacizumab	Double chemotherapy and monoclonal antibody
FOLFOX or FOLFIRI Cetuximab	Folinic acid 5FU Oxaliplatin or Irinotecan Cetuximab	Double chemotherapy and monoclonal antibody
FOLFOX or FOLFIRI Vectibix	Folinic acid 5FU Oxaliplatin or Irinotecan Vectibix	Double chemotherapy and monoclonal antibody
5FU platinum salts Pembrolizumab	5FU platinum salts Pembrolizumab	Double chemotherapy and immunotherapy
FOLFIRI ou FOLFOX Pembrolizumab	Folinic acid 5FU Oxaliplatin or Irinotecan Pembrolizumab	Double chemotherapy and immunotherapy
FOLFIRI or FOLFOX Nivolumab	Folinic acid 5FU Oxaliplatin or Irinotecan Nivolumab	Double chemotherapy and immunotherapy
FOLFIRI or FOLFOX Durvalumab	Folinic acid 5FU Oxaliplatin or Irinotecan Durvalumab	Double chemotherapy and immunotherapy
5FU Cisplatin Sintilimab	5FU Cisplatin Sintilimab	Double chemotherapy and immunotherapy
FLOT	5FU Oxaliplatin Docetaxel	Triple chemotherapy
FOLFIRINOX and mFOLFIRINOX	Folinic Acid 5FU Oxaliplatin Irinotecan	Triple chemotherapy
TFOX	Docetaxel 5 FU Folinique Acid Oxaliplatin	Triple chemotherapy
TPF	Docetaxel Cisplatin 5FU	Triple chemotherapy
FOLFIRINOX Bevacizumab	Folinic Acid 5FU Oxaliplatin Irinotecan Bevacizumab	Triple chemotherapy and monoclonal antibody
Spartalizumab FLOT	5FU Oxaliplatin Docetaxel Spartalizumab	Triple chemotherapy and immunotherapy

Treatment type was not correlated with DPD status in either univariate or multivariate analysis. Only age was associated with treatment type in multivariate analysis (*p* = 0.008).

### 3.3 DPD Status and 5FU Dosing

In the general population, the mean initial dose was 2070.76 mg/m^2^/daily. A priori dose reduction was performed in 32% of patients, mostly based on age and DPD status.

In univariate analysis, age (*p* = 0.011) and DPD status (*p* = 0.001) were both correlated with initial 5FU dose reduction. Starting dose was reduced by an average of −13% in PM individuals as compared with other patients (Table 3). Conversely, gender, performance status, BMI, disease stage, or renal impairment were not associated with dose reduction. On multivariate analysis, PM DPD status (OR = 0.2, [0.09; 0.44], *p* = 0.0001) and age (OR = 0.97, [0.95; 0.99], *p* = 0.0269) remained associated with dose reduction. The initial dose reduction was achieved by removing bolus 5FU, sometimes further combined with a reduction in the continuous infusion dose. Of note, some patients had further dose reductions in subsequent cycles regardless of DPD status and based solely on clinical tolerability, i.e., dose reductions after severe side effects or dose increases in patients who had excellent tolerability. Such subsequent dose modifications were statistically more frequent in PM patients (*p* = 0.027 ANOVA), with mean U values of 18 ng/mL, i.e., close to the threshold for DPD deficiency. This could explain why, although the initial dose was significantly different according to DPD status, the cumulative dose of 5FU was ultimately not statistically different among patients, despite a numerical difference towards lower cumulative dose in PM patients.

**TABLE 3:** 5FU doses and treatment modifications depending on DPD status.

	UM *N* = 42	PM *N* = 43	EM *N* = 267	*p*
Mean initial 5FU dosage (mg/m^2^/d) (± standard deviation)	2203.26 (±621.41)	1868.48 (±649.82)	2080.74 (±596.88)	0.006
Initial 5FU dosage reduction				<0.001
No	38 (90.5%)	0 (0%)	212 (79.4%)	
Yes	4 (9.5%)	43 (100%)	55 (20.6%)	
Mean number of cycles (± standard deviation)	7.5 (±4.45)	8.28 (±6.33)	8.04 (±5.33)	0.844
Dosage modification during 5FU treatment				0.012
No	35 (83.3%)	30 (69.8%)	228 (85.4%)	
Yes	7 (16.7%)	13 (30.2%)	39 (14.6%)	
Therapeutic pause				0.646
Yes	6 (14.3%)	4 (9.3%)	39 (14.6%)	
No	36 (85.7%)	39 (90.7%)	228 (85.4%)	
Cumulative IV 5FU dose (mg/m^2^) (± standard deviation)	20,213.75 ± 11,312.88	18,198.72 ± 12,949.59	22,106.89 ± 13,730.0	0.079
Treatment discontinuation due to severe toxicities	0	0	6 (2.07%)	Not tested

### 3.4 DPD Status and 5FU‐Related Toxicities

A total of 15 cases of severe toxicities were documented (4.8%; 4.3%; 4.7%, in UM, EM, and PM, respectively), including acute renal failure (*n* = 7); bone marrow failure (*n* = 4, none for UM); pulmonary embolism (*n* = 3); and intestinal obstruction (*n* = 1). We observed 16.2% grade 2 (9.5%; 16.2%; 23.2%, in UM, EM, and PM, respectively), and 79.5% grade 0–1 (85.7%; 79.5%; 72.1% in UM, EM, and PM, respectively). No statistically significant difference was observed in the severity of the reported adverse effects (*p* = 0.55, Chi‐squared test); however, UM did not exhibit any signs of digestive or haematological disorders (*p* = 0.018). No toxic death was encountered.

**FIGURE 1:**
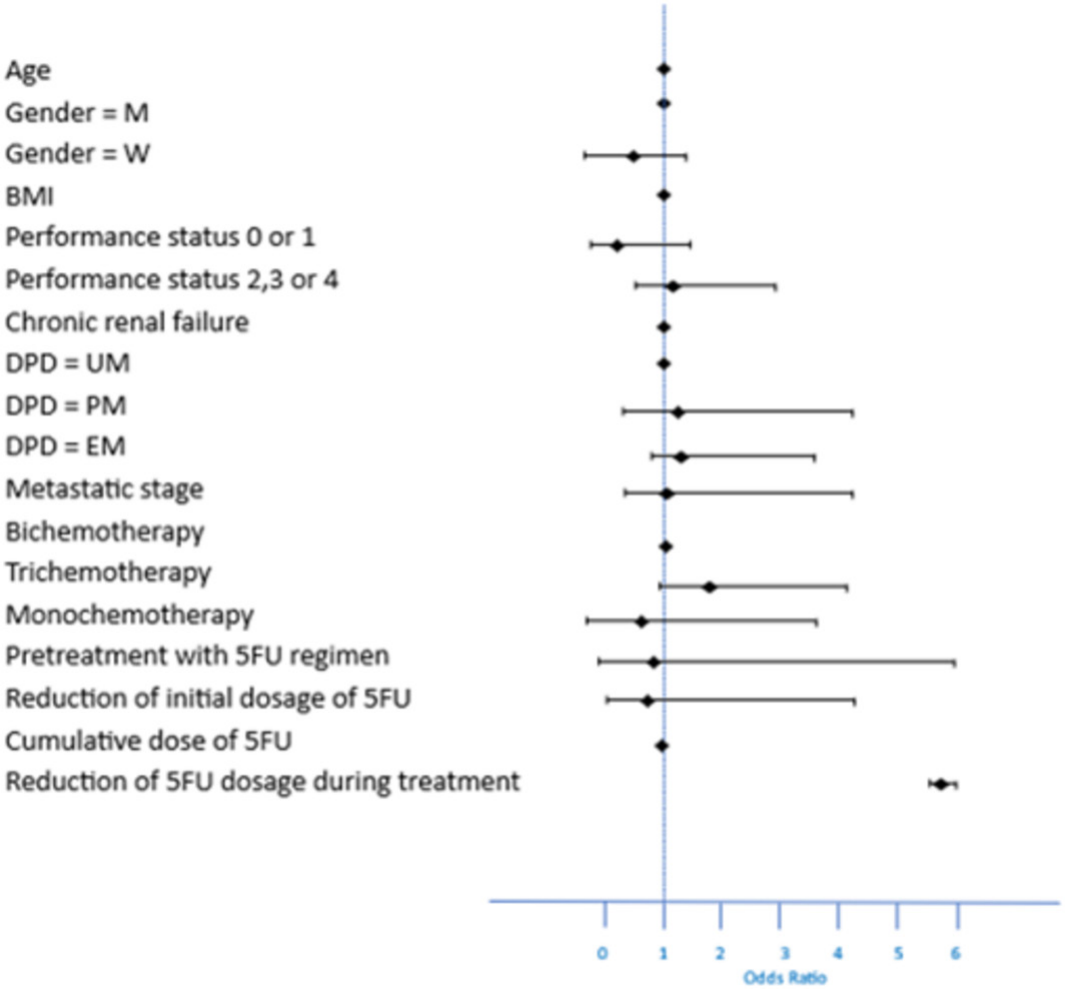
Factors identified as correlated with severe toxicity (multivariate analysis).

There was no difference in the total number of cycles administered based on DPD status (Table 3). In univariate and multivariate analysis (Figure 1), toxicity was associated with subsequent dose reduction (*p* < 0.0001). DPD status, gender, age, BMI, performance status, renal impairment, disease stage, pretreatment with 5FU, single agent, double or triple chemotherapy, initial dose of 5FU, and cumulative dose of 5FU were not predictive of severe toxicity. Of note, hematological toxicities (all grades) were only observed in PM and EM patients but not in UM patients. Similarly, digestive toxicities (all grades) were only observed in PM and EM patients and not in UM patients.

### 3.5 DPD Status and Treatment Efficacy

**FIGURE 2:**
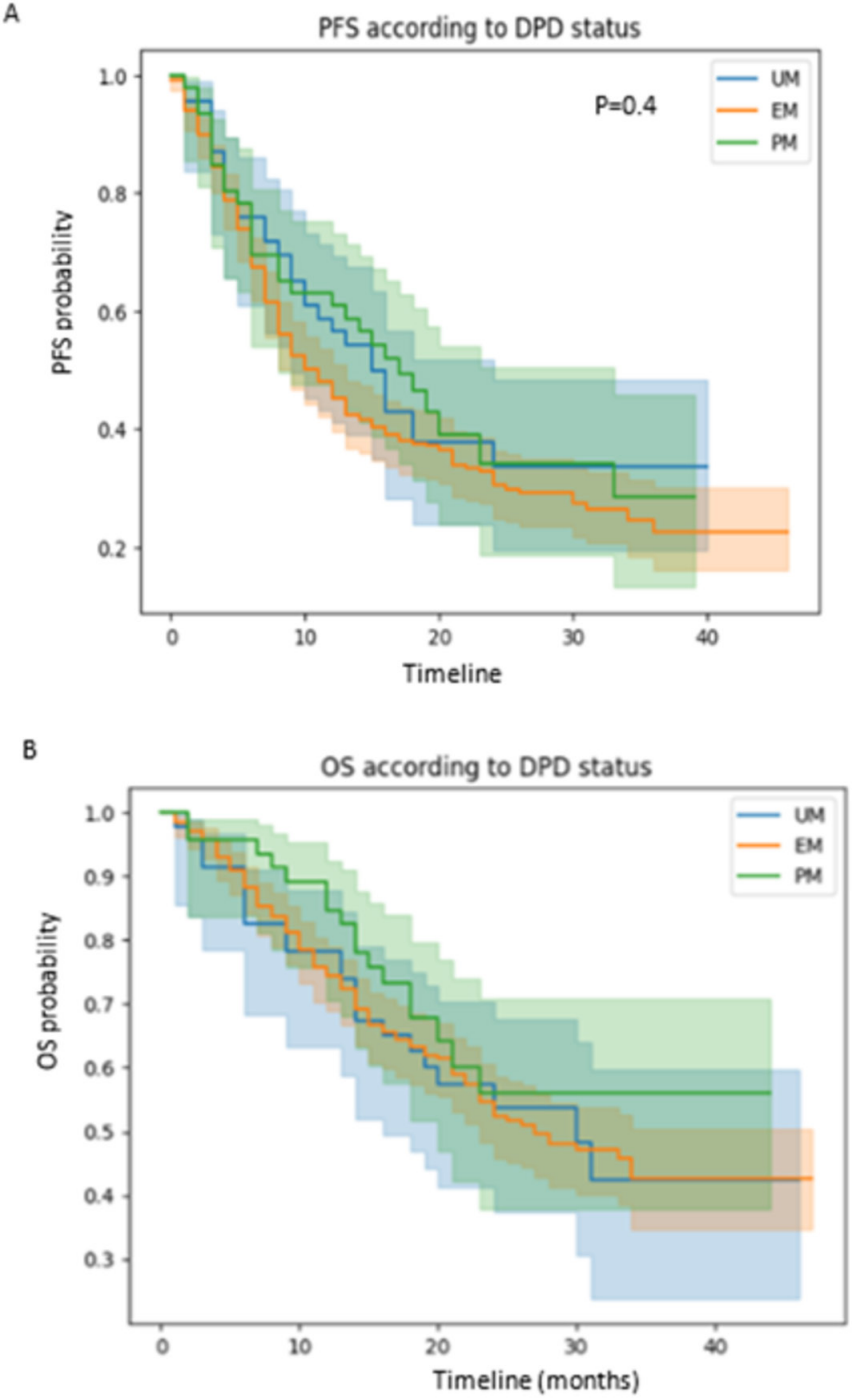
Progression‐free survival (PFS) (A) and overall survival (OS) (B) stratified by DPD status.

**FIGURE 3:**
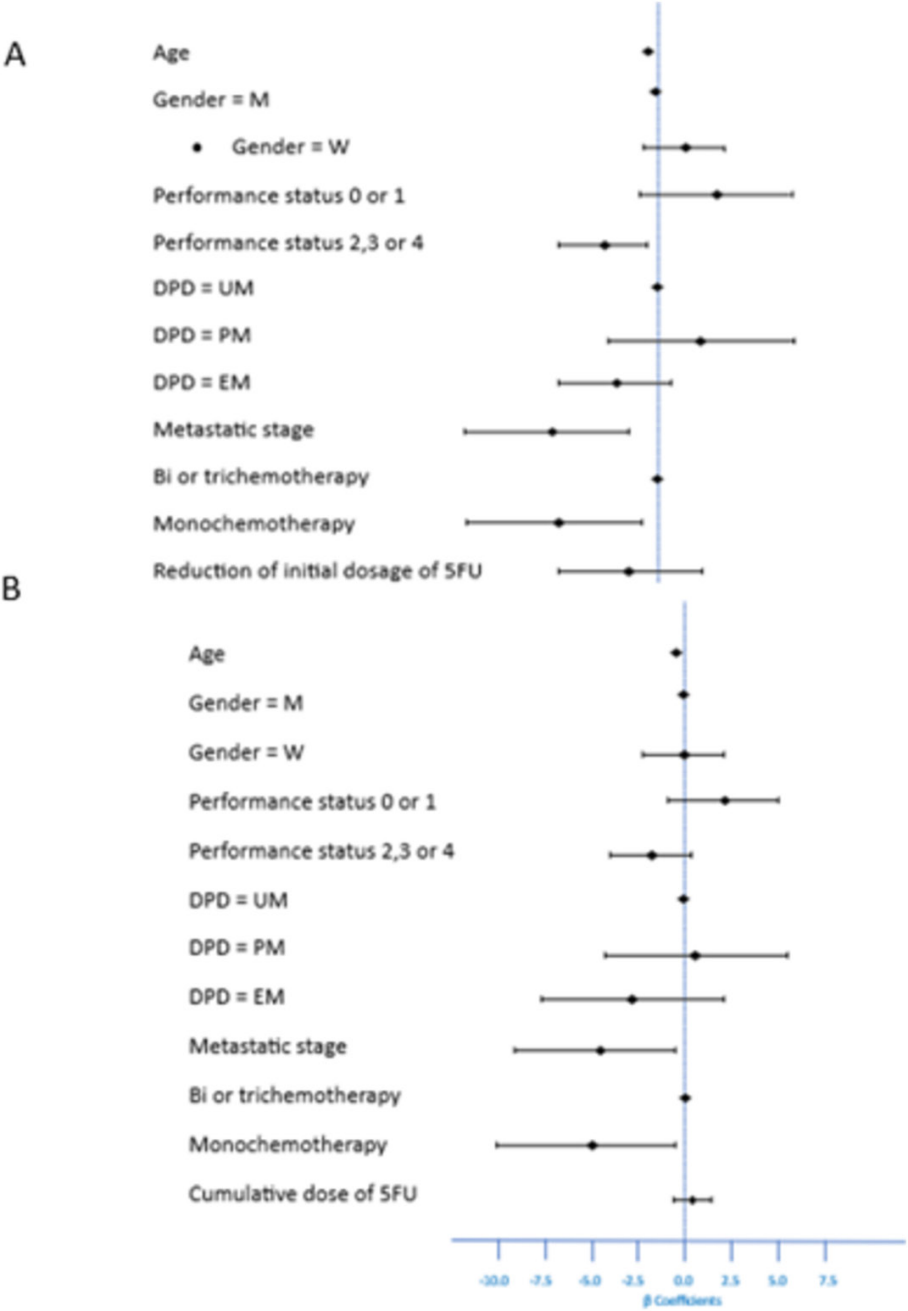
Factors influencing PFS (A) and OD OS (B) (multivariate analysis).

Mean follow‐up was 19.38 ± 10.25 months with no difference between groups according to DPD status. Mean PFS and OS were 13.37 and 25 months, respectively, in the overall population with no significant difference between the DPD groups (Figure 2). There was also no statistically significant difference in response to treatment (Table 4). Multivariate analysis showed no association between DPD status and Response Rate, PFS, or OS (Figure 3). For these three variables, a correlation was found only with disease stage (*p* = 0.00428 for treatment response; *p* = 0.008 for PFS; and *p* = 0.001 for OS). Age was correlated with both PFS and OS (*p* = 0.0142 for PFS; *p* = 0.0222 for OS), as was treatment with single‐agent chemotherapy (*p* = 0.026 for PFS; *p* = 0.0175 for OS). Performance status was statistically correlated with treatment response (*p* = 0.0431) and PFS (*p* = 0.0447). Gender and cumulative dose of 5FU were not associated with response, PFS, or OS.

**TABLE 4:** Efficacy depending on DPD status.

	UM *N* = 42	PM *N* = 43	EM *N* = 267	*p*
Response to treatment (CR, PR, SD)				0.596
Yes	24 (57.1%)	26 (60.4%)	141 (52.8%)	
No	18 (42.9%)	17 (39.6%)	126 (47.2%)	
Complete response				0.445
Yes	15 (35.7%)	18 (41.9%)	86 (32.2%)	
No	27 (64.3%)	25 (58.1%)	181 (67.8%)	
Median PFS (months) (± standard deviation)	16.3 (±10.25)	19.8 (±10.34)	12.3 (±10.7)	0.4
Progression‐Free at 18 months				0.374
Yes	18 (42.9%)	22 (51.2%)	89 (33.3%)	
No	24 (57.1%)	21 (48.8%)	178 (66.7%)	
Still alive at 18 months				0.875
Yes	26 (61.9%)	32 (74.4%)	156 (58.4%)	
No	16 (38.1%)	11 (25.6%)	111 (41.6%)	
Still alive at the end of follow‐up				0.281
Yes	21 (50%)	27 (62.8)	133 (49.8%)	
No	21 (50%)	16 (37.2)	134 (50.2%)	

Abbreviations: CR: complete response, PR: partial response, SD: stable disease, OR: objective response.

### 3.6 DPD Status and Clinical Outcome: A Focus in Patients With Colorectal Cancer (CRC)

When focusing on the more homogeneous subset of our cohort (i.e., 87 patients with CRC treated with double chemotherapy plus a monoclonal antibody), the results were consistent with those observed in the whole population about both the initial and cumulative doses of 5FU administered (Table 5). In the context of PM patients, the initial dose administered was found to be lower compared to other patients (1146.15 mg/m^2^/day vs. 1274.32 mg/m^2^/day, i.e., 11.8% decrease). A similar observation was made with the cumulative dose, which was found to be lower in the PM patient cohort (19 107 mg/m^2^ vs. 27 631 mg/m^2^, representing a 30% decrease). However, it is noteworthy that these numerical differences were not statistically significant (*p* = 0.061 and *p* = 0.079, respectively), possibly attributable to the limited sample size. The data also indicated that there were no differences in toxicities between patients with CRC depending on their DPD status (*p* = 0.08, Exact Fisher test). The proportion of patients experiencing adverse events of grade 0–2 was comparable, with 11.1% for UM, 25% for PM, and 18.4% for EM. Moreover, the occurrence of grade 3–4 adverse events was negligible (0% for UM vs. 0% for PM vs. 1.6% in EM). Similarly, no difference in efficacy was observed regarding DPD status. Median PFS was 10.22 months for UM, 10.9 months for PM, and 10.5 months for EM patients (*p* = 0.36 Log rank test). Median survival was not achieved by the overall population nor by any of the three distinct groups (*p* = 0.76 Log rank test).

**TABLE 5:** 5FU doses and treatment modifications depending on DPD status (patients with CRC).

	UM *N* = 9	PM *N* = 13	EM *N* = 65	*p*
Mean initial 5FU dosage (mg/m^2^/d) (± standard deviation)	1333.33 (±141.42) *N* = 9	1146.15 (±197.34) *N* = 13	1266.15 (±141.42) *N* = 65	0.061
Initial 5FU dosage reduction				0.71
No	7 (89.13%)	9 (6.52%)	43 (73.91%)	
Yes	2 (10.87%)	4(93.48%)	22 (26.09%)	
Mean number of cycles (± standard deviation)	8.55 (±2.92)	8.69 (±3.3)	11.13 (±2.9)	0.27
Further dosage modification during 5FU treatment				0.67
No	8 (89.13%)	9 (6.52%)	50 (73.91%)	
Yes	1 (10.87%)	4(93.48%)	15 (26.09%)	
Cumulative 5FU dose (mg/m^2^) (± standard deviation)	22,577.77 ± 8224.6	19,107.69 ± 7460.39	28,330.76 ± 9719.82	0.079
Treatment discontinuation due to severe toxicities	0	0	1 (1.54%)	Not tested

## 4 Discussion

5FU usually causes severe toxicities in 10% up to 30% of patients and fatalities in 0.1% to 1% of patients, depending on the regimen [19, 20]. Several reports have shown that most of the observed life‐threatening or lethal toxicities could be attributed to DPD deficiency [21]. Consequently, upfront testing for DPD deficiency has been made mandatory in France since 2018, using baseline U measurement as a surrogate for DPD activity [10, 22]. It is noteworthy that the UH2/U ratio was not retained by French health authorities to identify patients with DPD deficiency. While the metabolic ratio may appear more relevant, evidence has emerged indicating the superiority of isolated uracil as a better marker, particularly due to the inherent challenge of ensuring the reproducibility of ratio measurements [23]. For instance, when using standard UPLC‐UV techniques to measure analytes as in this work, the assay of UH2 should be done at 210 nm, a wavelength far from being specific, opening the way to multiple chromatographic interferences and overlapping peaks either with endogenous compounds or co‐medications, thus lowering the precision of assaying UH2. Using U alone as a surrogate, although rapid and convenient, has some drawbacks such as poor stability after sampling and uncertainties in the cut‐offs for intermediate/profound deficiency thresholds. To get rid of this uncertainty, we have also considered a higher cut‐off (i.e., 30 ng/mL) associated with PM status, but it did not change our results when comparing clinical outcomes between UM, EM, and PM patients eventually (data not shown). It should be noted that U and UH2 can also be measured by mass spectrometry (LC–MS/MS), which gives more robust results, especially for UH2; however, switching to LC–MS/MS does not solve the problems related to the poor stability of the analytes, nor the uncertainties in determining the appropriate cut‐offs as mentioned above. Most clinical studies have focused thus far on patients with an increased risk of toxicity due to partial or profound DPD deficiency [10]. There are only a few data on UM patients, who are considered at a lower risk of toxicity but could also be underdosed [9, 16]. Indeed, exposure–response relationships have been found for both toxicity and efficacy, and plasma AUC of 18–28 mg h/L is usually considered the target exposure with 5FU [23, 24]. In this study, UM patients represented 11.9% of patients treated with 5FU. Unlike deficiency, there is no clear consensus to identify a uracil plasma level associated with UM status. Because the median of U values was 10 ng/mL in our patients and the limit of quantification of uracil using our UPLC technique was 5 ng/mL, we empirically decided that U values < 5 ng/mL could be associated with UM status, based upon the hypothesis that the lower the uracil value, the higher the DPD activity.

Our data show that UM patients treated with standard dosing have the same efficacy as PM or EM patients. This observation differs from Chamorey et al., who found that UM patients treated at standard dosing had a shortened PFS [15]. Here, the variety of regimens and tumor localization could be a confounding factor. However, since UM, EM, and PM status were all equally distributed regardless of regimen and tumor localization, we believe that our data are meaningful. Also, when focusing on the homogenous subset of patients with CRC all treated with a double chemotherapy, we found similarly that DPD status had no influence on efficacy, thus supporting our global observation that UM status does not impair 5FU efficacy.

Baseline characteristics did not differ between UM, EM, and PM patients, suggesting that it is not possible to detect DPD deficiency if no upfront testing (i.e., genotyping or phenotyping) is made. There was no difference in the male‐to‐female ratio between the DPD groups, contrary to some previous studies suggesting that women may have a higher incidence of DPD deficiency [25, 26, 27]. However, those previous studies were based on *DPYD* genotyping [28, 29] or direct measurement of DPD activity in PBMC and not on uracilemia as here, a surrogate test that does not appear here to be influenced by patient age or sex. In addition, recent work suggests that patients with chronic renal insufficiency are more likely to be wrongly classified as PM, as assessed by plasma uracil measurement, due to possible impaired renal elimination of uracil [30]. However, another study has demonstrated that such a condition could be associated indeed with altered clearance of 5FU, and that elevated U values would still be representative of impaired elimination of the drug [13]. Here, although there was a trend toward more renal failure (i.e., eGFR < 60 mL/min/1.73m^2^) in PM patients in our study (i.e., 9.3% vs. 4.8% and 4.9% in PM, UM, and MS, respectively), this was not statistically significant. One explanation could be that most of our patients had only mild renal impairment (i.e., eGFR >45 mL/min/1.73m^2^), whereas it was suggested that severe renal failure was more likely to increase plasma uracil [29].

Additionally, there was no difference in the incidence of adverse events according to DPD status. The fact that PM patients did not experience more toxicities than patients with normal DPD activity highlights the efficacy of upfront adaptive dosing, as PM patients were treated with a −10% to −15% lower dose of 5FU than EM and UM patients, respectively. Of note, PM patients treated with reduced dosing showed the same response rate, PFS, and OS as other patients treated at standard dosing. It should be emphasized that the incidence of severe toxicities in our patients was remarkably lower than usually reported with 5FU, most likely due to the systematic initial dose reduction in PM patients. Consequently, the total number of cycles administered did not differ according to DPD status, suggesting that no more treatments were discontinued in PM patients than in other individuals, much probably due to upfront reduction in dosing lowering the risk of 5FU‐induced toxicities.

The main drawback of this study is the heterogeneity of settings and regimens. The fact that 5FU is rarely given as a single agent makes it difficult to avoid such confounding factors when discussing either efficacy or toxicity endpoints with this drug. In addition, the real‐world nature of this study means that no patients are censored as we present the raw efficacy and safety results of all patients treated with a 5FU‐containing regimen in our institution. In addition, the type of treatment did not appear to influence efficacy or toxicity on multivariate analysis, and when focusing on the subset of patients with CRC only, the same trends observed in the global cohort were found.

Overall, our data suggest that DPD UM patients have no compromised efficacy when treated with standard (i.e., mg/m^2^) 5FU. Therefore, there is no reason to increase a priori the dosing of 5FU in UM patients as they showed response rates, PFS, and OS similar to other patients. Additionally, the low incidence of severe toxicities we observed suggests that the upfront DPD testing with a priori reduction in 5FU dosage in PM patients helps to increase the safety of the 5FU‐containing regimen. Clear exposure‐response relationships have been identified with 5FU, as Area‐Under‐the‐Curve (AUC) comprised between 20 and 30 mg/L·h being considered as a target exposure with an acceptable toxicity–efficacy ratio. Associating pre‐emptive DPD testing with adaptive dosing in PM patients, plus therapeutic drug monitoring (TDM) of 5FU to check that the target AUC will be reached indeed could be an appealing strategy to further finely tune dosing in real‐time [31].

## 5 Conclusion

This real‐world study shows that UM patients are not at risk of compromised efficacy when treated with standard, BSA‐based dosing of 5FU. Additionally, we showed that the overall incidence of life‐threatening toxicities can be markedly reduced by performing upfront DPD testing with adaptive dosing in PM patients, thus contributing to increase the safety of this widely prescribed drug.

## Author Contributions

G.K. and J.C. designed the experiment. G.K., G.M., F.D., L.D. and J.C. collected and analyzed the data. G.K. G.M. and J.C. drafted the manuscript. All authors approved the manuscript. J.C. served as the guarantor of this work, thereby having complete access to all study data and assuming responsibility for the integrity and accuracy of the data analysis.

## Ethics Statement

The retrospective data collection and statistical analysis were approved by our Review Board (Cellule d'Evaluation at the Direction de la Recherche Clinique & de l'Innovation (DRCI), APHM) and followed the European recommendations regarding the Clinical Trials Regulation (EU) 536/20141 and the General Data Protection Regulation (GDPR; EU) 2016/6792.

## Consent

With respect to the non‐interventional, retrospective nature of this study, patients' written informed consent was waived by the Assistance Publique Hôpitaux de Marseille (APHM) review board.

## Conflicts of Interest

The authors declare no conflicts of interest.

## Data Availability

The data that support the findings of this study are available on request from the corresponding author. The data are not publicly available due to privacy or ethical restrictions.
